# The Association of Glucose Control with Circulating Levels of Red Blood Cell-Derived Vesicles in Type 2 Diabetes Mellitus Patients with Atrial Fibrillation

**DOI:** 10.3390/ijms24010729

**Published:** 2022-12-31

**Authors:** Alexander A. Berezin, Zeljko Obradovic, Kristen Kopp, Tetiana A. Berezina, Michael Lichtenauer, Bernhard Wernly, Alexander E. Berezin

**Affiliations:** 1 Zaporozhye Medical Academy of Postgraduate Education, 20 Vinter Av., 69096 Zaporozhye, Ukraine; 2 Klinik Barmelweid, Department of Psychosomatic Medicine and Psychotherapy, 5017 Barmelweid, Switzerland; 3 Department of Internal Medicine II, Division of Cardiology, Paracelsus Medical University of Salzburg, Strubergasse 21, 5020 Salzburg, Austria; 4 Department of Internal Medicine, Vita Center, 3 Sedov Str., 69000 Zaporozhye, Ukraine; 5 Department of Internal Medicine, General Hospital of Oberndorf, Paracelsusstraβe 37, 5110 Oberndorf bei Salzburg, Austria; 6 Center for Public Health and Healthcare Research, Paracelsus Medical University of Salzburg, Strubergasse 21, 5020 Salzburg, Austria; 7 Internal Medicine Department, Zaporozhye State Medical University, 26 Mayakovsky Av., 69035 Zaporozhye, Ukraine

**Keywords:** diabetes mellitus, heart failure, red blood cells, extracellular vesicles, hyperglycemia

## Abstract

Hyperglycemia is a trigger for structural alteration of red blood cells (RBCs) and their ability to release extracellular vesicles (EVs). The aim of the study was to elucidate whether glucose control in T2DM patients with concomitant HF and AF affects a circulating number of RBC-derived EVs. We prospectively included 417 T2DM patients with HF, 51 of them had atrial fibrillation and 25 healthy volunteers and 30 T2DM non-HF individuals. Clinical assessment, echocardiography examination and biomarker measures were performed at the baseline of the study. RBC-derived EVs were determined as CD235a+ PS+ particles by flow cytometry. NT-proBNP levels were measured by ELISA. AF patients with glycosylated hemoglobin (HbA1c) < 6.9% had lower levels of CD235a+ PS+ RBC-derived vesicles than those with HbA1c ≥ 7.0%. There were no significant differences in number of CD235a+ PS+ RBC-derived vesicles between patients in entire cohort and in non-AF sub-cohort with HbA1c < 6.9% and HbA1c ≥ 7.0%, respectively. Multivariate linear regression yielded that CD235a+ PS+ RBC-derived vesicles ≥ 545 particles in µL (OR = 1.06; 95% CI = 1.01–1.11, *p* = 0.044) independently predicted HbA1c ≥ 7.0%. Elevated levels of CD235a+ PS+ RBC-derived EVs independently predicted poor glycaemia control in T2DM patients with HF and AF.

## 1. Introduction

Heart failure (HF) and atrial fibrillation (AF) are common cardiovascular (CV) conditions, which frequently complicate type 2 diabetes mellitus (T2DM) and exert a combined detrimental impact on CV mortality and HF hospitalization, clinical status, exercise tolerance and quality of life [1]. Although HF continue to increase in prevalence as a leading risk factor for AF in general population [2,3], T2DM retains a powerful factor that intervenes in advancing adverse cardiac remodeling and risks of HF manifestation and AF occurrence regardless of age and gender of the patients [4,5]. Surprisingly, despite increasing prevalence of AF among patients with concomitant HF with reduced ejection fraction (HFrEF), its association with worse CV outcomes was found to be significant in patients with HF with mildly reduced (HFmrEF) and preserved (HFpEF) ejection fraction, but not in those with HFrEF [6]. Taking into consideration the fact that T2DM patients more often demonstrate HFpEF than HFrEF, AF may be a significant prognostic factor for diabetics with HFpEF/HFmrEF. Finally, HF and T2DM can exacerbate or be exacerbated by AF especially when they correspond to its persistence or permanent form [7]. Yet, AF is one of most important predictors of systemic thromboembolic complications among T2DM patients with any concomitant phenotypes of HF [8]. In patients with T2DM reactive oxygen species also increase the advanced glycation end products, which cause endothelial dysfunction and disrupt vascular homeostasis [9]. Increased protein kinase C activity causes increased endothelin-1 synthesis, leading to greater vasoconstriction and platelet aggregation [10]. Furthermore, protein kinase C activation modifies the nitric oxide signaling process and stimulates vasoconstriction. Finally, both insulin resistance and hyperglycemia contribute to the development of a pro-thrombotic state characterized by increased platelet activation and coagulation [11]. To update our knowledge, there was no strong evidence regarding a possibility to predict AF onset and occurrence of AF-related complications with a large number of conventional circulating biomarkers including brain natriuretic peptides [12].

Until now the underlying molecular mechanisms of diabetes-related AF is not fully understood, but they affect structural and electrical cardiac remodeling, electromechanical myocardial alteration, auto/paracrine dysregulation of cardiac and vascular, pre- and post-conditioning and hypercoagulation [13]. It is suggested that blood turbulence due to AF, as well as several conditions including HF and T2DM, mediate a release of various extracellular vesicles (EVs) in circulation from mother cells [14]. EVs are defined as a heterogenic lipid-bilayer sub-cellular group of particles with a range size of 30–4000 nm [15]. The majority of circulating EVs derived from red blood cells (RBC) and platelets, the role of them in thrombogenicity and AF-related complication is controversial [16]. Platelet-derived EVs have been recognized a biomarker of peripheral arterial thrombosis, whether RBC-derived EVs are able to carry pro-coagulant phospholipids such phosphatidylserine is regarded to be a mediator for systemic thrombosis and target organ damage [17]. Moreover, hyperglycemia enable to lose deformability of RBCs through elevated arginase activity and arginase I protein expression in RBCs and increase a content of pro-coagulant phospholipids in RBC-derived EVs [18]. Perhaps, altered RBCs via RBC-derived EVs may induce endothelial dysfunction and to increase cardiac injury during ischemia-reperfusion in T2DM [18,19]. However, there is a less known impact of glycaemia control in T2DM patients with concomitant HF and AF on a number of RBC-derived EVs. The aim of the study was to elucidate whether glucose control in T2DM patients with concomitant HF and AF affect a circulating number of RBC-derived EVs.

## 2. Results

### 2.1. General Patients’ Characteristics

The entire patient population composes of 417 patients (231 male, 55.4% and 186 female, 44.6%) with average age of 53 years (Table 1) as well as 25 healthy volunteers and 30 T2DM non-HF individuals. In T2DM group with concomitant HF body mass index (BMI) was 25.8 kg/m^2^, waist circumference was 95.1 cm and waist-to-hip ratio (WHR) was 0.85 units. Amongst most important co-existing conditions included hypertension (84.4%), dyslipidemia (83.0%), left ventricular (LV) hypertrophy (80.1%), smoking (40.3%), stable coronary artery disease [CAD] (33.8%) and chronic kidney disease [CKD] 1–3 grades (26.9%). Yet, mean fasting glucose concentration and HbA1c, circulating level of NT-proBNP were 6.12 mmol/L, 6.59%, and 2615 pmol/mL, respectively. The patients from entire patient population had I/II (67.6%) or III (32.4%) heart failure (HF) New York Heart Association (NYHA) class and were qualified as having HF with preserved (HFpEF), mildly reduced (HFmrEF) and reduced (HFrEF) ejection fraction in 31.7%, 33.6% and 34.8%, respectively. Average LV ejection fraction was 46% (95% CI = 37–54%). We did not find significant differences between AF and non-AF patients in demographics and anthropomorphic parameters, whereas profiles of comorbidities, HF phenotype presentation, HF NYHA functional classes and hemodynamics parameters sufficiently differed between both sub-cohorts. Indeed, dyslipidemia, stable CAD, LV hypertrophy and CKD 1–3 grades were detected more frequently in AF patients than among non-AF individuals. There were no statistically significant differences between these sub-cohorts in other comorbidities and CV risk factor presentation. Along with it, AF patients more often had HFrEF and III HF NYHA class than non-AF individuals, whereas last ones demonstrated higher proportion of HFpEF and I/II HF NYHA class than those with AF. LV end-diastolic (LVEDV) and end-systolic (LVESV) volumes, LV myocardial mass index (LVMMI), left atrial volume index (LAVI) and E/e′ ratio were significantly higher in AF persons when compared with non-AF patients. There were no differences in biomarkers profile between AF and non-AF patients apart from the levels of NT-proBNP, which were higher in those with AF than without it. Concomitant medications were prescribed according to a presence of AF and HF phenotype presentations and clearly demonstrate that only i/f blocker ivabradine has not been administered in AF individuals. However, non-AF patients had lower frequency of utilization of mineralocorticoid receptor antagonists (MRA) when compared with those who had AF.

Healthy volunteers matched T2DM non-NF patients in age, gender, anthropomorphic parameters, while WHR was significantly higher in those with T2DM than in healthy volunteers. However, four healthy volunteers only had smoking as a major CV risk factor. There were not significant changes in hemodynamics parameters between healthy volunteers and T2DM non-HF patients, whereas the levels of HOMA-IR, HbA1c, total cholesterol, low-density lipoprotein cholesterol (LDL-C) were found to be higher in T2DM non-NF patients than in healthy volunteers. T2DM non-HF patients were treated with metformin and some of them received ACE inhibitors (43.3%), calcium channel blockers (16.7%) and statins (80.0%). Finally, we noticed sufficient differences between T2DM patients with and without HF in WHR, presentation of several risk factors (hypertension, CAD, abdominal obesity, LV hypertrophy, CKD 1–3 grades), as well as in more increase in cardiac volumes and reduction in LVEF. Aline with it, HF T2DM patients had higher levels of NT-proBNP, creatinine, LDL-C, triglycerides, and lower values of eGFR than those without HF.

**Figure 1:**
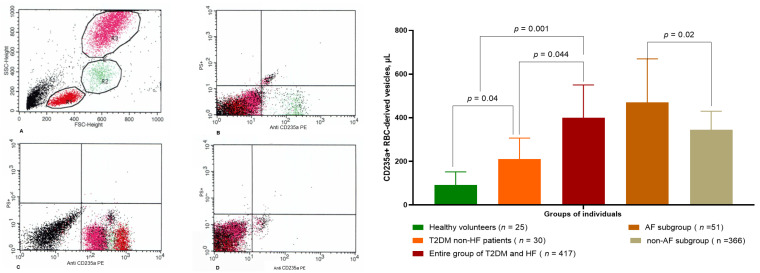
Amount of circulating CD235a+ PS+ RBC-derived vesicles: (**Left panel**) depicts results of flow cytometry measure of PS+ CD235a+ RBS-derived vesicles; Distinguishing populations of RBC-derived vesicles were determined at the first step of gaiting depending on their size (<0.5 μm) using forward scatter and side scatter dot plot (**A**). Next, CD235a+ RBC- derived vesicles and PS+ RBC- derived vesicles were determined as double-positive events for labels indicated as CD235a+ and PS+ in T2DM non-HF (**B**), T2DM patients with HF (**C**) when compared with healthy volunteers (**D**). (**Right panel**) illustrate comparable proportion of circulating amount of CD235a+ PS+ RBC-derived vesicles in different cohorts of eligible individuals. Abbreviations: T2DM, type 2 diabetes mellitus; HF, heart failure; AF, atrial fibrillation, RBC, red blood cells; PS, phosphatidylserine.

Circulating levels of CD235a+ PS+ RBCs-derived vesicles differed amongst T2DM patients depending on HF presentation when compared with healthy volunteers (Figure 1). Not surprisingly, the lowest amount of CD235a+ PS+ RBCs-derived vesicles (92 ± 60 particles in µL) were found in healthy volunteers. Meanwhile, there were the patients from other groups had higher levels of ones. However, average entity of CD235a+ PS+ RBCs-derived vesicles in T2DM individuals without HF was 210 ± 97 particles in µL, whereas in T2DM individual with HF it was 402 ± 153 particles in µL. We noticed statistical difference between the levels of CD235a+ PS+ RBCs-derived vesicles in AF and non-AF-subgroups (470 ± 199 particles in µL and 345 ± 85 particles in µL, respectively, *p* = 0.02).

### 2.2. Amount of CD235a+ PS+ RBC-Derived Vesicles in T2DM Patients with HF Depending of Glycemia Control

**Figure 2:**
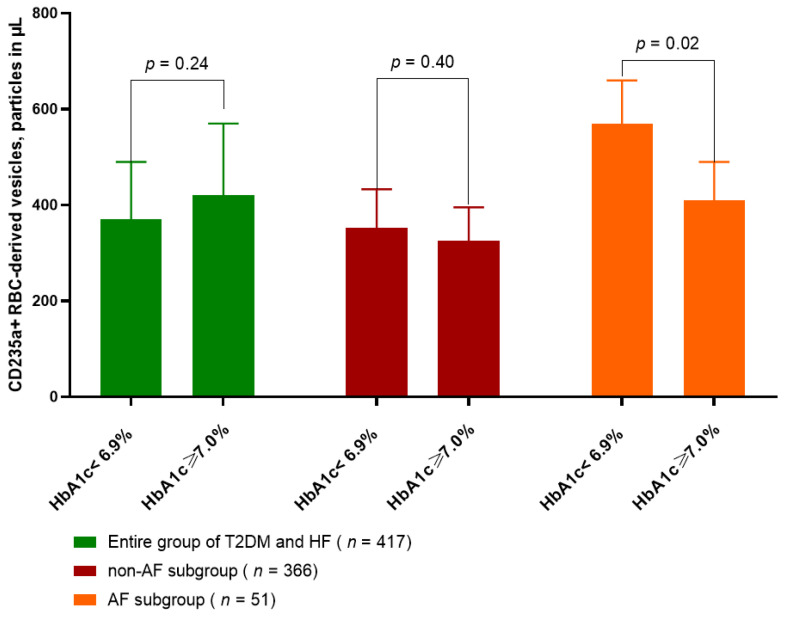
The amount of CD235a+ PS+ RBC-derived vesicles in T2DM patients with HF depending of glycemia control. Abbreviations: T2DM, type 2 diabetes mellitus; HF, heart failure; AF, atrial fibrillation, RBC, red blood cells; PS, phosphatidylserine; HbA1c, glycosilated hemoglobin.

Figure 2 illustrated the differences in circulating amount of CD235a+ PS+ RBC-derived vesicles in AF and non-AF patients with T2DM and HF depending on glycemia control, which were defined as HbA1c < 6.9 % and ≥7.0%. We established that among T2DM patents with HF and AF significantly lower levels of CD235a+ PS+ RBC-derived vesicles were detected in those with HbA1c < 6.9% (410 ± 80 particles in µL) than in patients with HbA1c ≥ 7.0% (574 ± 92 particles in µL). To note, there were no significant differences in circulating amount of CD235a+ PS+ RBC-derived vesicles between patients in entire cohort (370 ± 120 particles in µL and 420 ± 150 particles in µL, *p* = 0.24) and in non-AF subcohort (325 ± 70 particles in µL and 353 ± 80 particles in µL, *p* = 0.40) with HbA1c < 6.9% and HbA1c ≥ 7.0%, respectively. 

### 2.3. Spearman’s Correlation between Quantity of CD235a+ PS+ RBC-Derived Vesicles and Other Patients Characteristics

In a group of T2DM patients without HF amount of CD235a+ PS+ RBC-derived vesicles were positively associated with fasting glucose (r = 0.32, *p* = 0.01), and negatively correlated with LDL-C (r = −0.32, *p* = 0.04). We did not find any correlations between amount of CD235a+ PS+ RBC-derived vesicles and HbA1c, BMI, hemodynamic parameters including office blood pressure, LVEF, E/e′ as well as CV risk factors and NT-proBNP.

In entire group of T2DM patients with HF amount of CD235a+ PS+ RBC-derived vesicles correlated positively with HbA1c (r = 0.32, *p* = 0.001), creatinine (r = 0.30, *p* = 0.02), fasting glucose (r = 0.30, *p* = 0.01), and negatively correlated with eGFR (r = −0.28, *p* = 0.01).

Among AF subgroup there were positive associations between amount of CD235a+ PS+ RBC-derived vesicles with HbA1c (r = 0.42, *p* = 0.001), LAVI (r = 0.41, *p* = 0.001), NT-proBNP (r = 0.36, *p* = 0.001), LVMMI (r = 0.39, *p* = 0.001), BMI (r = 0.36, *p* = 0.001), creatinine (r = 0.32, *p* = 0.01), and fasting glucose (r = 0.30, *p* = 0.01). On the contrary, in non-AF subgroup there were positive mild association with HbA1c (r = 0.26, *p* = 0.01) and fasting glucose (r = 0.22, *p* = 0.01), but not with hemodynamic parameters and lipids. We also did not find correlation between circulating amount of CD235a+ PS+ RBC-derived vesicles and age, gender, a number of CV risk factors and HF phenotypes in any groups of eligible patients.

### 2.4. The Predictive Ability of Amount of CD235a+ PS+ RBC-Derived Vesicles for Poor Glycaemia Control: The Receive Operation Characteristics Curve Analysis

**Figure 3:**
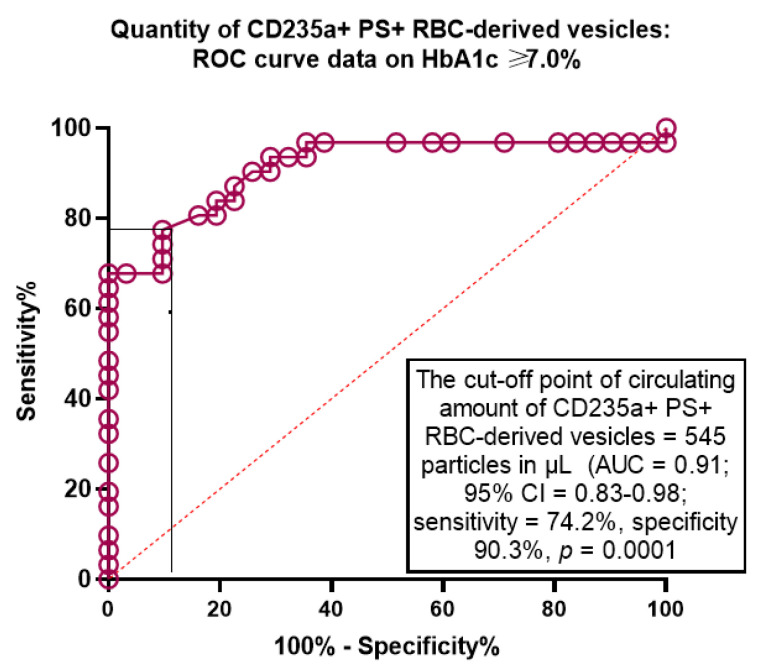
The predictive ability of circulating amount of CD235a+ PS+ RBC-derived vesicles for poor glycaemia control The Receive Operation Characteristics curve analysis. Abbreviations: AUC, area under curve; CI, confidence interval; RBC, red blood cells; PS, phosphatidylserine.

The Receive Operation Characteristics curve analysis revealed that the well-balanced cut-off point for circulating amount of CD235a+ PS+ RBC-derived vesicles (HbA1c ≥ 7.0% versus HbA1c < 6.9%) were 545 particles in µL (area under curve [AUC] = 0.91, sensitivity = 74.2%, specificity = 90.3%; *p* = 0.0001) (Figure 3).

### 2.5. The Predictors of Poor Glycemic Control in T2DM Patients with HF: The Univariate and Multivariate Linear Regression

In order to identify plausible value of circulating amount of CD235a+ PS+ RBC-derived vesicles as predictor of poor glycemic control we used the univariate linear regression model (Table 2). We established that HFrEF (OR = 1.04; 95% CI = 1.02–1.07, *p* = 0.042), LAVI (OR = 1.05; 95% CI = 1.03–1.09, *p* = 0.044), NT-proBNP (OR = 1.07; 95% CI = 1.03–1.12, *p* = 0.01) and CD235a+ PS+ RBC-derived vesicles ≥ 545 particles in µL (OR = 1.05; 95% CI = 1.02–1.09, *p* = 0.01) predicted HbA1c ≥ 7.0%. Multivariate linear regression yielded that NT-proBNP (OR = 1.07; 95% CI = 1.02–1.10, *p* = 0.04) and CD235a+ PS+ RBC-derived vesicles ≥ 545 particles in µL (OR = 1.06; 95% CI = 1.01–1.11, *p* = 0.044) remained independent predictors for HbA1c ≥ 7.0%.

### 2.6. The Comparisons of Predictive Models for HbA1c ≥ 7.0%: The Results of Model Fit Statistics

We did not find significant differences between the predictive ability of two models constructed from NT-proBNP and CD235a+ PS+ RBC-derived vesicles, while combined use of them allowed reclassifying patients with HF and adding discriminatory information (Table 3).

## 3. Discussion

The results of our study showed that circulating levels CD235a+ PS+ RBC-derived EVs increased depending on AF presentation in T2DM patients with HF and that poor glycaemia control was associated with elevated levels of CD235a+ PS+ RBC-derived EVs. Perhaps, these finding can shed a light on causes of previously established deference in CV mortality between patients with and without AF having any phenotypes of HF [6,19]. Indeed, HF and AF have many of the same risk factors including age and T2DM and stem from highly resembling pathophysiological derangements, such as myocardial ischemia and apoptosis with loss of cardiac myocytes, endothelial dysfunction and reperfusion damage, accumulating extracellular matrix and microvascular inflammation [20]. In fact, hemodynamic parameters including LAVI and LVEF were reported to be practically useful predictors for HF in AF independently from the conventional risk factors, whereas higher ventricular heart rate was no longer associated with higher mortality in HFpEF [21,22]. On the contrary, these parameters did not reproduce strong specificity to predict AF-related complications in HF patients with metabolic comorbidity including T2DM [23]. Indeed, AF-related outcomes in HF patients can be a result of HF progression due to worsening adverse cardiac remodeling, any target organ damages, such as T2DH-induced chronic kidney disease (CKD), systemic embolism and arterial thrombosis [24]. Therefore, forms of AT can intervene in clinical event. Indeed, among HF patients with AF history, those with paroxysmal AF had greater risk of CV outcomes including HF hospitalization and stroke than individuals with persistent or permanent AF [25]. This association considerably reflects underlining importance of anticoagulant therapy, which did not sustainably provide patients at first with paroxysm of AF [26]. On the other hand, data received from national registry “Outcomes Registry for Better Informed Treatment of Atrial Fibrillation”, which included 10,135 AF patients with HF, yielded a strict similarity in rates of thromboembolic complications regardless of LVEF among patients with any forms of AF [27].

In this context, a role of conventional circulating biomarkers as predictors of AF-related events appears to be controversial. There is a large amount of evidence regarding the fact that elevated levels of NT-proBNP had similar predictive value for adverse CV outcomes, irrespective of AF status, in HFrEF as well as in non-HF patients with different forms of AF [28,29]. However, this issue does not relate HFpEF individuals with and without AF who frequently had T2DM. Indeed, among patients with NT-proBNP ≥ 400 pg/mL, the relationship between NT-proBNP and CV outcomes differs with lower absolute risk in AF patients in comparison with those who do not have any forms of AF [30]. Along with it, T2DM increased risks of new-onset and recurrent HF-related outcomes, CV and all-cause mortality in patients with any AF independently from NT-proBNP and CV risk factor signature [31]. However, an impact of glycemic control in risk modification remained uncertain. 

We obtained results, which demonstrated that circulating levels CD235a+ PS+ RBC-derived EVs, which composed of pro-coagulant lipids, were closely associated with poor glycemic control in AF HF individuals with T2DM regardless of the levels of NT-proBNP. We suggested that the underlying mechanisms by which both biomarkers reflected higher risk are different. Elevated levels of NT-proBNP in AF patients tackled closer with fluid overload, cardiac volume increase and reduced LVEF than circulating pool of CD235a+ PS+ RBC-derived EVs. On the contrary, there are numerous findings, which undoubtedly clarify that CD235a+ PS+ RBC-derived EVs are involved in accelerating atherosclerosis and thrombosis, regulation of vascular tone, mediating systemic and microvascular inflammation and coagulation [32,33]. Another interesting suggestion is based on the idea that hemoglobin, which is composed of RBC-derived EVs, might be involved in NO-scavenging and thereby impair the endothelial-dependent vasodilation [34].

It has not to be surprised, when RBC-derived EVs are found in elevated concentration in patients with metabolic syndrome and early-to-late stages of T2DM [35]. However, until now the majority of data revealed that there were no remarkable differences between the levels of RBC-derived EVs in healthy volunteers and T2DM patients who were treated with aspirin, antidiabetic drugs, but not insulin [36,37]. In this case, we first reported that among well-treated T2DM patients with concomitant HF regardless of their AF status the levels of CD235a+ PS+ RBC-derived EVs was sufficiently higher than in healthy volunteers and T2DM without HF and AF. Thus, T2DM is considered powerful cause directly leading to elevated amount of CD235a+ PS+ RBC-derived EVs, which links hyperglycemia with cardiac and vascular damage. Moreover, regardless of HF treatment in accordance with contemporary guideline-based approach poor glycemic control supported elevated number of these particles in circulating blood. One of possible mechanisms, by which this effect became possible, is hyperglycemia-induced oxidative stress and mitochondrial damage, which are regarded to be crucial in accumulation of advanced glycation end products, active radicals, low-oxidized lipids [38]. These molecules are involved in epigenetic regulation of transcriptional processes through methylation of target substrates in mitochondria, ATP-dependent chromatin modeling and histone modification along with a regulation of micro-RNAs synthesis [39,40]. All these underlying pathways are regarded to be involved in altered deformation of RBCs, decrease in their membrane stability, ability to release EVs embarked by pro-coagulant components [41]. Consequently, RBCs micro-vesiculation and persistence of pro-coagulant CD235a+ PS+ RBC-derived EVs in, respectively, high concentrations in peripheral blood is a result of complex pathological processes, which are under control of pro-inflammatory genes, epigenetic regulation, endocytosis by endothelial cells, and mechanical impact of altered intra-cardiac blood flow, systemic inflammation and atherosclerosis [42,43,44]. This can explain why current anti-diabetic therapy did not influence critical reduction in phosphatidylserine-exposing RBC-derived EVs and why poor glycemic control continues to be such a dramatic risk factor for HF patients with T2DM. In addition to that, CD235a+ PS+ RBC-derived EVs may play a pro-coagulative role in AF patients, because in non-ST elevation myocardial infarction patients this biomarker predicted a higher risk of in-stent thrombosis after percutaneous coronary intervention [45]. It is possible that pharmacological blockade of phosphatidylserine could become a novel target for translation therapy for the prevention of CV outcomes in AF patients. 

Finally, we suggested that our results might open new perspectives for stratification of T2DM patients with any phenotypes of HF at risk of AF and AF-related events. Future investigations can include patients after ablations and hybrid procedure (ablation and pacing) for whom a prediction of recurrent AF continues to be crucial. However, CD235a+ PS+ RBC-derived EVs demonstrated a possibility to improve predictive value of NT-proBNP and this finding is considered extremely important for patients with low levels of the pro-peptide during treatment of HF. The next step in further investigation is a validation of the model and obtain clear information about economic burden of the use of this measure.

The study has several limitations. First, we routinely included patients with established persistent or permanent forms of AF, while patients with paroxysmal form of AF were not included in AF subgroup until recovered sinus rhythm continued to be supported. Next, limitation was associated with low number of healthy volunteers and T2DM non-HF subjects to be used for comparison with the T2DM patients with confirmed AF. This study was initially designed as an investigation among HF patients with T2DM regardless of their AF status. Thus, AF was detected as concomitant condition in entire group of eligible patients. Along with it, we included small proportion of AF patients after ablation procedure for whom new episode(s) of AF was determined as post-procedural recurrent AF. We did not analyze hemodynamics and electrophysiological parameters in this group. However, serial measures of circulating amount CD235a+ PS+ RBC-derived EVs during administration of antidiabetic drugs and improving glucose control were not performed. However, we suggest these limitations will not have a crucial impact on data interpretation.

## 4. Materials and Methods

### 4.1. Study Design and Cohorts of Participants

**Figure 4:**
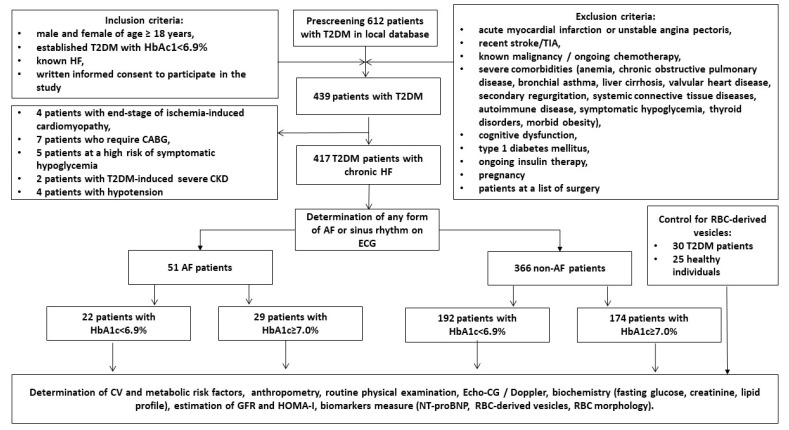
Study design and patients’ procedure flow chart. Abbreviations: AF, atrial fibrillation; CABG, coronary artery bypass grafting; CV, cardiovascular; CKD, chronic kidney disease; GFR, glomerular filtration rate; HF, heart failure; HbA1c, glycated hemoglobin; HOMA-IR, Homeostatic Assessment Model of Insulin Resistance; N-terminal brain natriuretic pro-peptide; T2DM, type 2 diabetes mellitus; TIA, transient ischemic attack.

A total of 612 patients with T2DM from the local database of the private hospital Vita-Centre (Zaporozhye, Ukraine) were prescreened and then invited to participate in the study. Any sources of medical data including medical records, discharge reports, laboratory reports, were used to identify candidates for this study. However, personal communications with general practitioners to enroll patients in the study according to inclusion/exclusion criteria was performed (Figure 4).

As inclusion, age ≥ 18 years, T2DM, established HF, and written consent to participate in the study was used. Patients after successful ablation procedure (mainly radiofrequency ablation) were included in the group non-AF in 6 weeks after procedure if cardiac rhythm is sinus. Exclusion criteria were acute coronary syndrome/myocardial infarction or unstable angina pectoris, recent stroke/transient ischemic attack, known malignancy, severe co-morbidities (anemia, chronic lung and liver diseases, known inherited and acquired heart defect, symptomatic severe hypoglycemia, morbid obesity, systemic connective tissue diseases, autoimmune disease, cognitive dysfunction and thyroid disorders), pregnancy, type 1 diabetes mellitus or current therapy with insulin. In the end, we enrolled 417 individuals with T2DM who had chronic HF and subdivided them into two groups depending on criteria of poor glycemic control (HbA1c < 6.9% and ≥7.0%, respectively). Study protocol was described in detail in the article published [46].

### 4.2. Determination of Anthropometric Parameters, Co-Morbidities and Concomitant Diseases 

Conventional anthropometric parameters including height, weight, waist circumference, hip-to-waist ratio (WHR), body mass index (BMI) and body surface area (BSA) were measured according to current recommendations [47]. Microalbuminuria was defined as albumin urine excretion in the range of 30–299 mg/g creatinine [48]. T2DM and HF was established according to conventional clinical recommendations [49,50]. Poor glycaemia control was referred as HbA1c ≥ 7.0% [50]. The European Society of Cardiology (ESC) clinical guidelines were used to determine concomitant disease and CV risk factors, such as hypertension [51], dyslipidemia [52], coronary artery disease/chronic coronary syndrome [53]. Chronic kidney disease in T2DM patients was detected in accordance with Kidney Disease Improving Global Outcomes (KDIGO) Consensus Report [54].

### 4.3. Examination of Hemodynamics

B-mode echocardiography and Doppler examination of the patients was performed by blinded ultra-sonographer with the diagnostic system Vivid T8 (“GE Medical Systems”, Freiburg, Germany) in compliance with current guidelines [55,56]. Left ventricular end-diastolic (LVEDV), end-systolic (LVESV) volumes, and left atrial volume (LAV) were sampled in the apical 4-chamber and/or 5-chamber views and LAV index (LAVI) was calculated as a ratio of LAV to BSA. Left ventricular (LV) ejection fraction (LVEF) was measured by the Simpson method. Diastolic parameters included early diastolic blood filling (E), longitudinal strain ratio (e′) and their ratio (E/e′). Estimated E/e′ ratio was expressed as the ratio equation of E wave velocity to averaged medial and lateral e’ velocity [55]. Left ventricular hypertrophy (LVH) was detected when the LV myocardial mass index (LVMMI) was ≥125 g/m^2^ or ≥110 g/m^2^ in male and female, respectively [56].

### 4.4. Diet and Medications

Eligible T2DM patients received a personal lifestyle modification program and dietary recommendation along with adjusted dose of metformin and/or sodium-glucose cotransporter-2 (SGLT2) inhibitor. Other concomitant medications included renin-angiotensin-aldosterone blockers, mineralocorticoid receptor antagonists and beta-blockers. Loop diuretics, i/f blocker ivabradine, lipid-lowering agents, antiplatelet drugs and anticoagulants were prescribed when needed.

### 4.5. Blood Sampling, Measurement of Circulating Biomarkers and Determination of Glomerular Filtration Rate and Insulin Resistance

Fasting blood samples from patients were collected from an antecubital vein (3–5 mL) and maintained at 4 °C. After centrifugation (3000 r/min, 30 min) polled serum aliquots were immediately stored at ≤–70 °C until analysis. Serum concentrations of NT-proBNP were determined using commercially available enzyme-linked immunosorbent assay (ELISA) kits (Elabscience, Houston, TX, USA) according to the manufacturer’s instructions. All ELISA data were analyzed according to the standard curve and each sample was measured in duplicate as the mean value was finally analyzed. Both the intra- and inter-assay coefficients of variability for each biomarker were <10%. 

Conventional biochemistry parameters were routinely measured at the local biochemical laboratory of Vita-Centre (Zaporozhye, Ukraine) using a Roche P800 analyzer (Basel, Switzerland). We used CKD-EPI formula to estimate glomerular filtration rate (GFR) [57]. Insulin resistance was evaluated using the Homeostatic Assessment Model of Insulin Resistance (HOMA-IR) estimation [58].

### 4.6. Isolation, Detection and Quantitation of Circulating CD235a+ PS+ RBC-Derived EVs

The blood samples for RBC-EVs quantitation were pooled in previously cooled silicon tubes with 3.2% solution of sodium citrate. Centrifugation protocol included preliminary centrifugation of diluted sample at 300× *g* during 15 min and 2000× *g* during 20 min at room temperature to remove living and dead cells [59]. Since then, cell-free supernatant was removed by aspiration and immediately transferred into cooled silicon tubes to be centrifuged at 25,000× *g* to pellet EVs of the typical size with further removing. Next, a portion of cell-free supernatant was additionally centrifuged at 100,000× *g* during 25 min to pellet RBC-derived EVs. Centrifugation was performed on analytical ultracentrifuge Optima (Beckman Coulter, Germany). Pellets received after each centrifugation step were re-suspended in Milli-Q ultra-pure water. Enriched in RBC-derived EVs samples were collected and further analyzed by flow cytometry technique [60].

Flow cytometry was performed according to conventional protocol with FMO standards to check a noise using standard calibration kit (BD Calibrite™, BD Calibrite 3 Beads, BD Biosciences) with evaluation of 5000 events/s [60]. In order to detect RBC-derived EVs we used the following fluorochrome coupled antibodies: anti-C235 FITC (REAfinity, Miltenyi Biotec, Auburn, CA, USA) and phosphatidylserine (PS) Alexa Fluor 488 (Creative Diagnostics, Shirley, NY, USA). The parameters of the gate window were calibrated to identify events less than 0.5 μm. Forward scatter and side scatter dot plot was re-presented on two-colour fluorescence histograms. Double-positive events for these labels characterized CD235a+ PS+ RBC-derived EVs. The analysis was performed by certified flow cytometry specialist. We used commercial flow cytometer Beckman CytoFlex S (Beckman Coulter, Germany) and the quantitation of CD235a+ PS+ RBC-derived EVs (particles in µL) was executed using the FCS Express 4 (DeNovo Software, Glendale, CA, USA).

### 4.7. Statistics

Statistical analysis was executed using SPSS 11.0 for Windows and v. 9 GraphPad Prism (GraphPad Software, San Diego, CA, USA). Continuous variables were expressed as means ± SD for parametric data and median and interquartile range [IQR] according to whether they were normally distributed or not. Kolmogorov–Smirnov test was used to test for normal distribution. The distribution of dichotomous values was analyzed with Chi-square test. We performed One-way Analysis of Variance (ANOVA) and Tukey test for comparisons of variables between subgroups. Spearman coefficient was used for correlations. The predictive ability of circulating number of CD235a+ PS+ RBC-derived vesicles for poor glycaemia control was evaluated using the Receive Operation Characteristics (ROC) curve analysis. We analyzed area under curve (AUC), confidence interval (CI), sensitivity and specificity as well as detected optimal cutoff point for circulating quantity of CD235a+ PS+ RBC-derived vesicles with the Jouden index. Predictors of poor glycemic control were determined by univariate and multivariate linear regression analysis. We reported odds ratio (OR), 95% confidence interval (95% CI) for each variable included in regression analysis. Predictive value of amount of CD235a+ PS+ RBC-derived vesicles for poor glycemic control was reclassified using the integrated discrimination indices (IDI) and net reclassification improvement (NRI). Differences were considered significant at the level of statistical significance *p* < 0.05.

## 5. Conclusions

We established that elevated levels of CD235a+ PS+ RBC-derived EVs independently predicted poor glycaemia control in T2DM patients with HF and AF regardless of NT-proBNP. Along with it, CD235a+ PS+ RBC-derived EVs were able to add more predictive information to model constructed on NT-proBNP. These findings may open new perspectives to use CD235a+ PS+ RBC-derived EVs in T2D patients with HF in stratification at risk AF and perhaps to recognize these pro-coagulant particles as target to be serially evaluated. New studies are required in the future to clearly elucidating the role of a dynamic changes of CD235a+ PS+ RBC-derived EVs in peripheral blood during anti-diabetic agent administration and improving glycemic control.

## Figures and Tables

**Table 1:** Baseline general characteristics of eligible T2DM patients compared with healthy volunteers and T2DM non-HF patients.

Variables	Healthy Volunteers (*n* = 25)	T2DM Non-HF Patients (*n* = 30)	T2DM HF Patients					
			p * Value	Entire Patient Cohort (*n* = 417)	p ** Value	AF Patients (*n* =51)	Non-AF Patients (*n* = 366)	p *** Value
Demographics and anthropomorphic parameters								
Age, year	51 (47–55)	52 (47–55)	0.88	53 (41–64)	0.88	55 (44–66)	51 (40–64)	0.06
Male/female *n* (%)	14 (56.0)/11 (44.0)	17 (57.0) 13 (43.0)	0.80	231 (55.4)/186 (44.6)	0.76	31 (60.7)/20 (39.3)	200 (54.6)/166 (45.4)	0.05
BMI, kg/m^2^	23.9 ± 2.7	24.1 ± 2.3	0.82	25.8 ± 2.8	0.80	26.0 ± 2.7	25.5 ± 2.9	0.92
Waist circumference, cm	86.1 ± 3.6	88.2 ± 3.0	0.78	95.1 ± 3.2	0.04	96.4 ± 3.0	94.6 ± 2.7	0.78
WHR, units	0.73 ± 0.04	0.84 ± 0.05	0.04	0.85 ± 0.05	0.88	0.87 ± 0.04	0.85 ± 0.06	0.76
Comorbidities and CV risk factors								
Dyslipidemia, *n* (%)	-	24 (80.0)	0.001	346 (83.0)	0.86	48 (94.1)	298 (81.4)	0.02
Hypertension, *n* (%)	-	13 (43.3)	0.001	352 (84.4)	0.01	42 (82.3)	310 (84.7)	0.88
Stable CAD, *n* (%)	-	-	-	141 (33.8)	0.001	26 (51.0)	115 (31.4)	0.01
Smoking, *n* (%)	4 (16)	11 (36.7)	0.001	168 (40.3)	0.001	23 (45.1)	145 (39.6)	0.14
Abdominal obesity, *n* (%)	-	9 (30.0)	0.001	179 (42.9)	0.01	20 (39.2)	159 (43.4)	0.12
LV hypertrophy, *n* (%)	-	7 (23.3)	0.001	334 (80.1)	0.001	47 (92.2)	287 (78.4)	0.01
CKD 1–3 grades, *n* (%)	-	4 (13.3)	0.001	112 (26.9)	0.001	19 (37.2)	93 (25.4)	0.04
Microalbuminuria, *n* (%)	-	5 (16.7)	0.001	75 (18.0)	0.66	8 (15.7)	67 (18.3)	0.10
HF phenotypes and functional classification								
HFpEF, *n* (%)	-	-	-	132 (31.7)	0.001	12 (23.5)	120 (32.6)	0.04
HFmrEF, *n* (%)	-	-	-	140 (33.6)	0.001	16 (31.3)	124 (33.9)	0.80
HFrEF, *n* (%)	-	-	-	145 (34.8)	0.001	23 (45.1)	122 (33.3)	0.01
I/II HF NYHA class, *n* (%)	-	-	-	282 (67.6)	0.001	29 (59.9)	253 (69.1)	0.04
III HF NYHA class, *n* (%)	-	-	-	135 (32.4)	0.001	22 (43.1)	113 (30.9)	0.01
Hemodynamics parameters								
SBP, mm Hg	124 ± 5	129 ± 7	0.82	132 ± 7	0.72	135 ± 5	129 ± 6	0.86
DBP, mm Hg	73 ± 4	76 ± 5	0.80	78 ± 5	0.80	79 ± 7	77 ± 5	0.86
LVEDV, mL	141 (122–155)	144 (126–158)	0.86	162 (154–170)	0.01	169 (159–180)	161 (152–169)	0.04
LVESV, mL	52 (44–61)	55 (47–64)	0.87	86 (80–93)	0.01	94 (86–99)	84 (80–89)	0.02
LVEF, %	63 (59–68)	62 (56–67)	0.88	46 (37–55)	0.02	44 (35–52)	48 (40–56)	0.05
LVMMI, g/m^2^	102 ± 4	108 ± 5	0.74	154 ± 5	0.001	170 ± 7	151 ± 5	0.04
LAVI, mL/m^2^	31 (29–34)	34 (30–36)	0.80	43 (37–52)	0.001	48 (41–56)	40 (36–45)	0.01
E/e′, unit	6.45 ± 0.3	6.51 ± 0.4	0.82	13.5 ± 0.3	0.001	14.9 ± 0.3	12.8 ± 0.2	0.01
Biomarkers								
eGFR, mL/min/1.73 m^2^	109 ± 5.5	102 ± 4.5	0.90	75 ± 4.0	0.001	72 ± 3.6	76 ± 6.0	0.80
HOMA-IR	4.32 ± 0.7	5.95 ± 0.9	0.01	7.95 ± 2.3	0.44	8.02 ± 2.2	7.90 ± 2.5	0.88
Fasting glucose, mmol/L	5.1 ± 0.7	6.08 ± 0.8	0.01	6.12 ± 1.3	0.72	6.50 ± 1.4	5.80 ± 1.5	0.84
HbA1c, %	5.20 ± 0.04	6.40 ± 0.05	0.01	6.59 ± 0.02	0.76	7.12 ± 0.10	6.42 ± 0.09	0.72
Creatinine, µmol/L	69.5 ± 7.0	77.4 ± 8.0	0.78	108.6 ± 8.5	0.01	110.4 ± 9.4	104.8 ± 8.9	0.84
TC, mmol/L	4.93 ± 0.50	5.48 ± 0.40	0.02	6.43 ± 0.60	0.04	6.50 ± 0.72	6.26 ± 0.50	0.88
HDL-C, mmol/L	1.04 ± 0.12	1.01 ± 0.15	0.88	0.97 ± 0.17	0.74	0.92 ± 0.20	0.99 ± 0.13	0.89
LDL-C, mmol/L	2.88 ± 0.13	3.10 ± 0.14	0.01	4.38 ± 0.10	0.02	4.60 ± 0.11	4.20 ± 0.12	0.76
TG, mmol/L	1.70 ± 0.10	1.80 ± 0.12	0.86	2.21 ± 0.17	0.01	2.28 ± 0.10	2.15 ± 0.15	0.68
NT-proBNP, pmol/mL	48 (10–95)	56 (0–102)	0.88	2615 (1380–3750)	0.001	3620 (1240–4250)	2190 (1170–3250)	0.02
Concomitant medications								
ACEI, *n* (%)	-	13 (43.3)	0.001	198 (47.5)	0.78	23 (45.1)	175 (47.8)	0.86
ARB, *n* (%)	-	-	-	67 (16.1)	0.001	6 (11.7)	61 (16.7)	0.16
ARNI, *n* (%)	-	-	-	165 (39.6)	0.001	20 (39.2)	145 (39.6)	0.92
Beta-blocker, *n* (%)	-	-	-	372 (89.2)	0.001	51 (100.0)	321 (87.7)	0.05
Ivabradine, *n* (%)	-	-	-	59 (14.1)	0.001	0	59 (14.1)	0.001
Calcium channel blocker, *n* (%)	-	5 (16.7)	0.001	75 (18.0)	0.42	8 (15.7)	67 (18.3)	0.05
MRA, *n* (%)	-	-	-	283 (67.8)	0.001	39 (76.5)	244 (66.7)	0.04
Loop diuretic, *n* (%)	-	-	-	358 (85.9)	0.001	43 (84.3)	315 (86.1)	0.90
Antiplatelet, *n* (%)	-	-	-	367 (88.0)	0.001	38 (74.5)	329 (89.9)	0.04
Anticoagulant, *n* (%)	-	-	-	51 (12.2)	0.001	51 (12.2)	0	0.001
Metformin, *n* (%)	-	30 (100)	0.001	387 (92.8)	0.80	45 (88.2)	342 (93.4)	0.02
Statins, *n* (%)	-	24 (80.0)	0.001	408 (97.8)	0.01	49 (96.1)	359 (98.1)	0.92

Notes: data of variables are given mean ± SD and median (25–75% interquartile range), p* value, a difference between healthy volunteers and T2DM non-HF patients; p** value, a difference between T2DM non-HF patients and T2DM HF patients; p*** value, a difference between values in AF and non-AF patients. Abbreviations: ACEI, angiotensin-converting enzyme inhibitor; angiotensin receptor neprilysin inhibitor (ARNI); CAD, coronary artery disease; CKD, chronic kidney disease; BMI, body mass index; DBP, diastolic blood pressure; E/e′, early diastolic blood filling to longitudinal strain ratio; GFR, glomerular filtration rate; HDL-C, high-density lipoprotein cholesterol; HFpEF, heart failure with preserved ejection fraction; HFmrEF, heart failure with mildly reduced ejection fraction; HFrEF, heart failure with reduced ejection fraction; LVEDV, left ventricular end-diastolic volume; LVESV, left ventricular end-systolic volume; LVEF, left ventricular ejection fraction; LVMMI, left ventricle myocardial mass index, left atrial volume index, LAVI; left atrial volume index; LDL-C, low-density lipoprotein cholesterol; MRA, mineralocorticoid receptor antagonist; SBP, systolic blood pressure; TG, triglycerides; TC, total cholesterol; WHR, waist-to-hip ratio.

**Table 2:** The predictors for poor glycemic control in study population. The results of univariate and multivariate logistic regression analysis adjusted to HOMA index and BMI.

Variables	Dependent Variable: HbA1c ≥ 7.0%			
	Univariate Linear Regression		Multivariate Linear Regression	
	OR (95% CI)	*p*-Value	OR (95% CI)	*p*-Value
III NYHA class vs I/II NYHA class	1.03 (1.00–1.07)	0.050	-	
AF versus sinus rhythm	1.08 (0.93–1.17)	0.82	-	
HFrEF vs HFpEF/HFmrEF	1.04 (1.02–1.07)	0.042	1.05 (1.01–1.09)	0.050
LAVI	1.05 (1.03–1.09)	0.044	1.03 (1.00–1.07)	0.050
NT-proBNP	1.07 (1.03–1.12)	0.010	1.07 (1.02–1.10)	0.040
CD235a+ PS+ RBC-derived vesicles ≥ 545 particles in µL vs. <545 particles in µL	1.05 (1.02–1.09)	0.040	1.06 (1.01–1.11)	0.044

Abbreviations: AF, atrial fibrillation, RBC, red blood cells; PS, phosphatidylserine; NT-proBNP, N-terminal brain natriuretic pro-peptide; HFrEF, heart failure with reduced ejection fraction; HFpEF, heart failure with preserved ejection fraction; HFmrEF, heart failure with mildly reduced ejection fraction; LAVI, left atrial volume index; NYHA, New York Hear Association; OR, odds ratio.

**Table 3:** The comparisons of NT-proBNP and CD235a+ PS+ RBC-derived EVs discriminative potencies for poor glycemic control.

Models	AUC		NRI		IDI	
	M (95% CI)	*p*-Value	M (95% CI)	*p*-Value	M (95% CI)	*p*-Value
NT-proBNP	0.66 (0.60–0.74)	-	Reference	-	Reference	-
CD235a+ PS+ RBC-derived vesicles	0.91 (0.83–0.98)	0.001	0.45 (0.39–0.52)	0.001	0.49 (0.44–0.55)	0.001

Abbreviations: AUC, area under curve; NT-proBNP, N-terminal brain natriuretic pro-peptide; CI, confidence interval; M, median; IDI, integrated discrimination indices; NRI, net reclassification improvement.

## Data Availability

Not applicable.
